# Detailed Visualization of Phase Evolution during Rapid Formation of Cu(InGa)Se_2_ Photovoltaic Absorber from Mo/CuGa/In/Se Precursors

**DOI:** 10.1038/s41598-018-22214-y

**Published:** 2018-03-02

**Authors:** Jaseok Koo, Sammi Kim, Taehoon Cheon, Soo-Hyun Kim, Woo Kyoung Kim

**Affiliations:** 1 School of Chemical Engineering, Yeungnam University, Gyeongsan, Gyeongbuk 38541 Republic of Korea; 2 Daegu Gyeongbuk Institute of Science & Technology, Dalseong, Daegu 42988 Republic of Korea; 3 School of Materials Science and Engineering, Yeungnam University, Gyeongsan, Gyeongbuk 38541 Republic of Korea

## Abstract

Amongst several processes which have been developed for the production of reliable chalcopyrite Cu(InGa)Se_2_ photovoltaic absorbers, the 2–step metallization–selenization process is widely accepted as being suitable for industrial–scale application. Here we visualize the detailed thermal behavior and reaction pathways of constituent elements during commercially attractive rapid thermal processing of glass/Mo/CuGa/In/Se precursors on the basis of the results of systematic characterization of samples obtained from a series of quenching experiments with set-temperatures between 25 and 550 °C. It was confirmed that the Se layer crystallized and then melted between 250 and 350 °C, completely disappearing at 500 °C. The formation of CuInSe_2_ and Cu(InGa)Se_2_ was initiated at around 450 °C and 550 °C, respectively. It is suggested that pre-heat treatment to control crystallization of Se layer should be designed at 250–350 °C and Cu(InGa)Se_2_ formation from CuGa/In/Se precursors can be completed within a timeframe of 6 min.

## Introduction

It has been a number of decades since chalcopyrite Cu(InGa)Se_2_ (CIGS) thin films were thoroughly explored as a potential candidate for use as light absorbers in high–efficiency thin film photovoltaic cells. Recent results have achieved a cell efficiency of 22.8%^1^, which is comparable and even superior to multi-crystalline Si cells. Amongst several processes which have been developed for the production of reliable CIGS absorbers^2–4^, the 2–step metallization–selenization process is widely accepted as being suitable for industrial-scale application. For example, the rapid thermal processing (RTP) of Se-coated Cu/Ga/In metal precursors was successfully scaled-up by the Avancis company (later managed by Saint–Gobain), reporting record efficiencies of 17.5% for a 30 × 30 cm^2^ mini–module and 13.9% for fully-integrated 65 × 165 cm^2^ modules^4,5^. However, it should be mentioned that the mini- and full-sized module efficiencies (13.9~17.5%) are still significantly lower than the highest CIGS cell efficiency that has been obtained (22.8%), partially due to the lack of a detailed understanding of the RTP mechanism for CuGa/In/Se type precursors. Further improvement in the conversion efficiency and reliability of CIGS absorbers fabricated by RTP from Mo/CuGa/In/Se precursors may require the optimization of many parameters such as the Cu/Ga/In metal stacking order, Se layer thickness, temperature ramp rate, annealing temperature, reaction time, partial pressure of Se.

One of the major issues present in a 2–step process, such as metallization and selenization, is the poor adhesion of CIGS to the Mo layer. This is presumably due to volume expansion, caused by a reaction of Se with the CuGaIn precursor to form a chalcopyrite CIGS structure^6^. Abou–Ras *et al*.^7^ suggested that MoSe_2_ layers grown parallel to the Mo surface could also cause poor adhesion at the Mo/CIGS interface, a theory which is now widely accepted^8–11^. Solid selenium can exist in several forms, including amorphous, hexagonal, α-monoclinic and β-monoclinic structures^12^. In the precursor structure of Mo/Cu–Ga–In/Se it is believed that the crystallization of amorphous Se layer is accompanied by significant volume change, subsequently resulting in mechanical stress of the metal precursor^13,14^. This subsequently results in poor adhesion of the CIGS layer to Mo, as evidenced by the delamination of CIGS layers from Mo after selenization and/or sulfurization. In our recent paper^15^, the results of *in-situ* high–temperature X–ray diffraction (HT–XRD; PANalytical X’pert Pro MPD) on CuGa/In/Se precursors with a temperature ramp rate of 0.33 °C/s (~20 °C/min) revealed that the initially amorphous Se layer in the CuGa/In/Se precursor structure would begin to crystallize at around 120 °C, then liquefy at its melting point of c.a. 220 °C. The intermediate phase of CuSe_2_ formed at 290 °C and the formation of CuInSe_2_ (CIS) and CIGS phases were initiated at 280 °C and 350 °C, respectively. In this paper, the detailed thermal behavior and reaction pathways of constituent elements during RTP of CuGa/In/Se precursors have been visualized based on the results of a systematic characterization of samples obtained from a series of quenching experiments. Particular attention was given to observing the behavior of Se and the intermixing of Ga–In.

## Result and Discussion

To investigate the detailed behaviour of the precursors during ramp up to the target temperature, a series of quenching experiments were performed. The precursors were heated to the target temperature of 550 °C at a fixed ramp up rate of 4 °C/s. The first run was stopped immediately after the thermocouple read 220 °C and the sample was quenched. Following a similar pattern, the next runs were intentionally halted at 50 °C increments up to a maximum of 550 °C. In addition, time-resolved isothermal annealing was conducted at 250 and 550 °C.

### 25 °C (as–deposited precursor)

**Figure 1:**
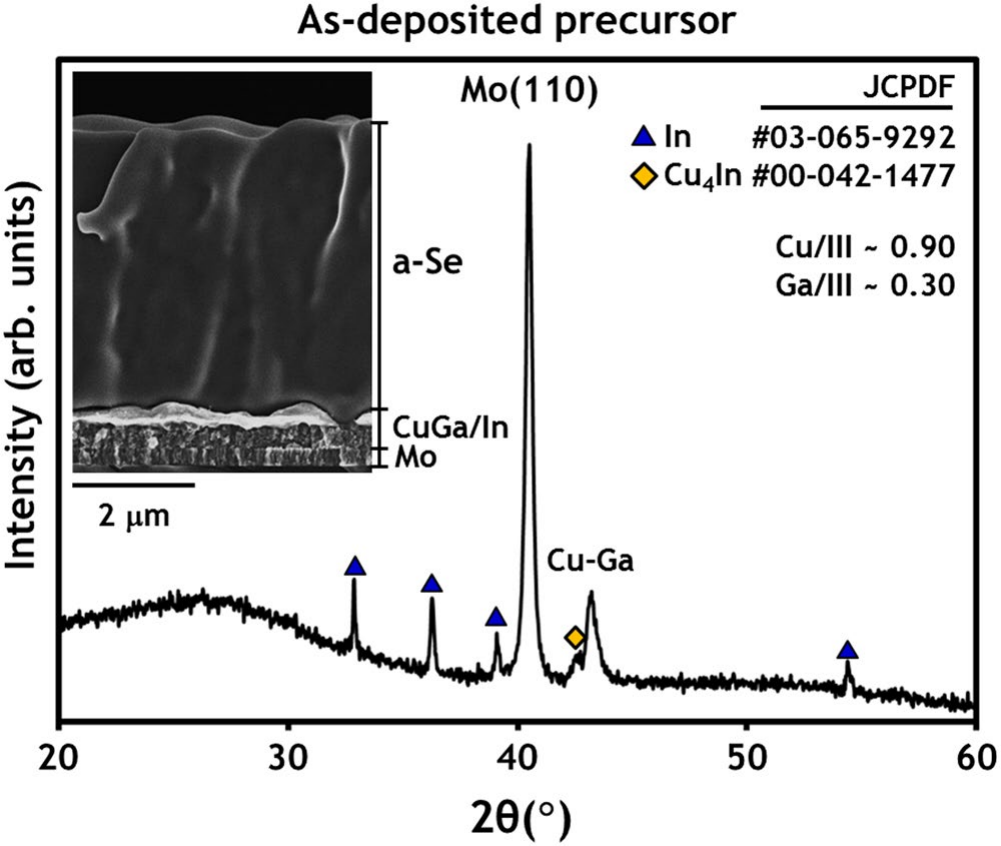
XRD scan result and SEM image of as-deposited glass/Mo/CuGa/In/Se precursors.

XRD analysis and field emission scanning electron microscopy (FE-SEM) cross-sectional image of the as-deposited CuGa/In/Se precursor confirm that it consists of pure In metal and intermetallics (e.g., Cu–Ga and Cu_4_In), with a 5 μm-thick Se layer as shown in Fig. 1. It should be noted that the Se layer deposited by vacuum evaporation, without heating of the substrate, has an amorphous phase structure. As is typically the case for sputter-deposited bilayer CuGa/In samples, the metallic CuGa/In layer is likely to have a pure In (or In–rich) nodule layer on the surface of a CuGa(In) intermetallic layer due to the thermodynamic immiscibility of a Cu-Ga-In mixture. Inductively coupled plasma–atomic emission spectroscopy (ICP-AES; Shimadzu ICPS-8100) analysis revealed the bulk compositions of samples to be Cu/III ~0.9 and Ga/III ~0.3, as was intended by the experimental design.

### 25 → 200 → 250 °C

**Figure 2:**
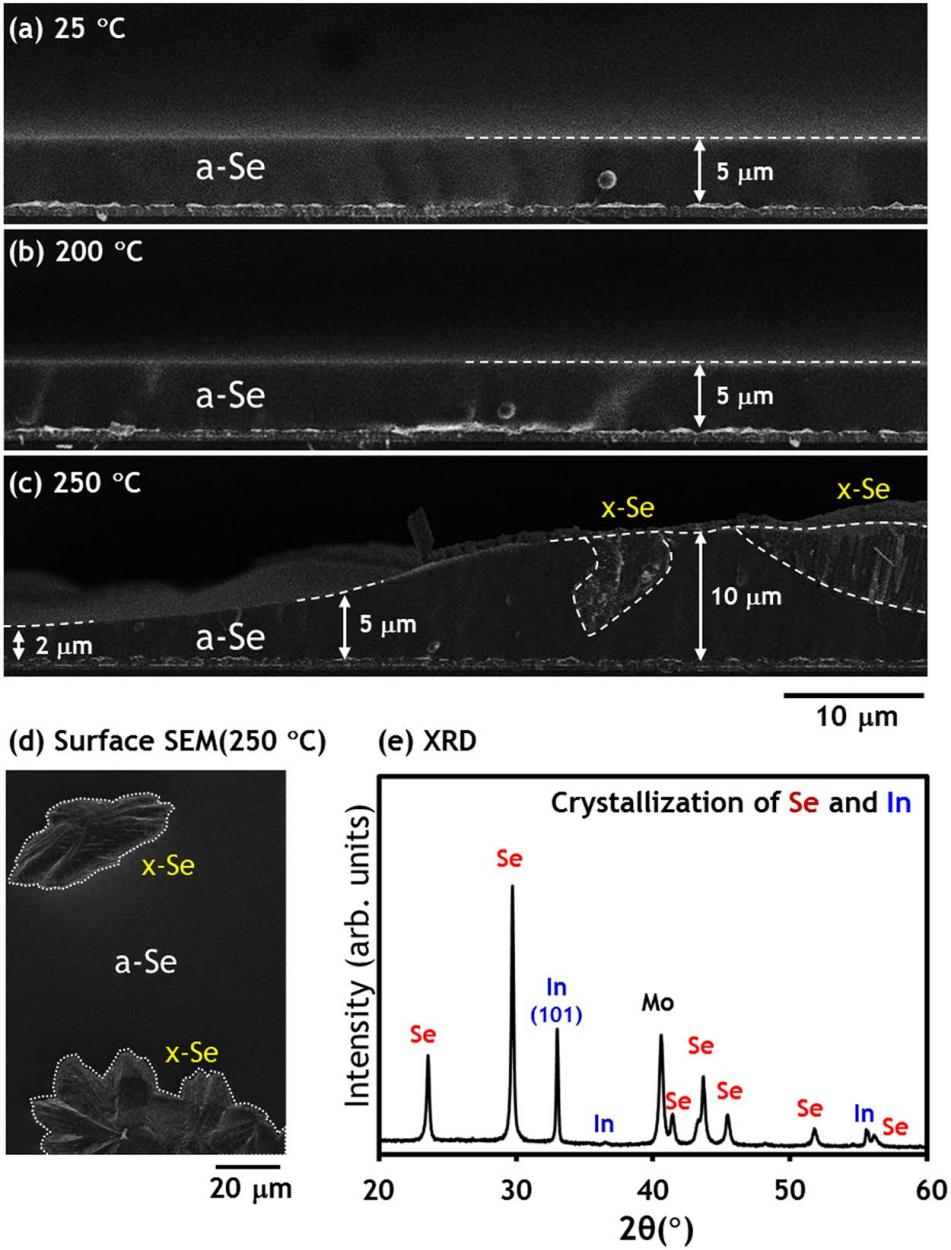
SEM images and XRD scan results of precursor and samples quenched from 200 °C and 250 °C. (**a**) As-deposited glass/Mo/CuGa/In/Se precursor with 5 μm-thick amorphous Se layer, (**b**) sample quenched from 200 °C and (**c**–**e**) sample quenched from 250 °C.

Figure 2 shows that there is no significant change in the amorphous Se layer until reaching 200 °C. As evidenced by Fig. 2(c) and (d), the partial crystallization of the Se layer and subsequent uplift of the crystallized region, were clearly observed in the 250 °C−quenched samples. The results of XRD analysis in Fig. 2(e) also confirmed further crystallization and/or texturization of In in the (101) direction, as well as crystallization of amorphous Se. It is interesting to recall that the crystallization of the Se layer during *in-situ* HT-XRD analysis^15^ of an identical precursor (glass/Mo/CuGa/In/Se), with a lower ramp rate of 0.33 °C/s, is initiated at around 120 °C, which should be closer to the thermodynamic equilibrium. Another set of quenching experiment with different cooling rate, i.e., quenching (~−2.4 °C/s) and slower cooling (~−0.55 °C/s) from 250 °C, revealed that slower cooling yielded more crystallization of Se evidenced by stronger intensity and smaller FWHM (0.128° with −0.55 °C/s vs. 0.154° with −2.4 °C/s for Se(101)) of Se XRD peaks (Supplementary Figure S1) as well as SEM cross-sectional images (Supplementary Figure S2).

### 250 °C (Isothermal annealing)

**Figure 3:**
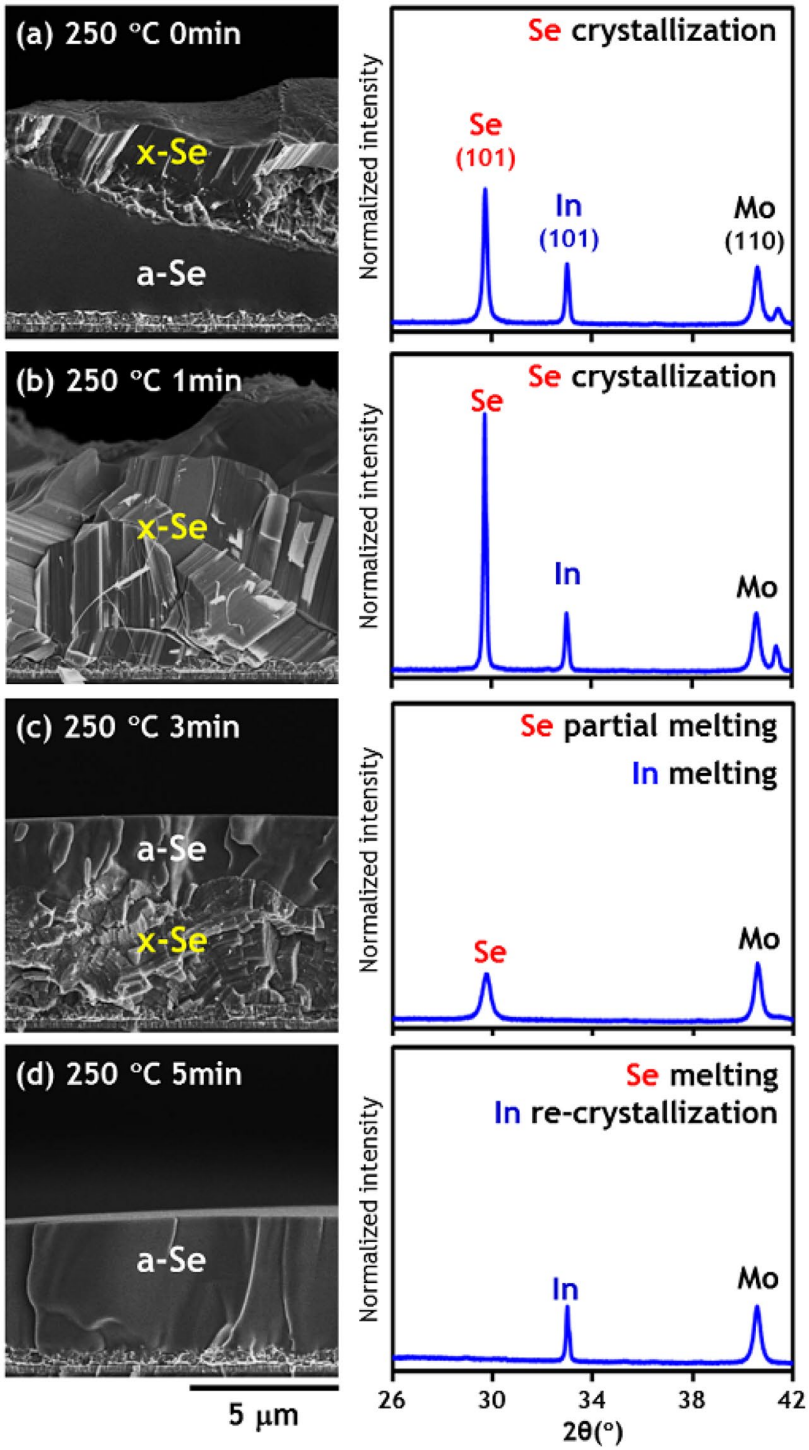
SEM images and XRD scan results of samples quenched from isothermal annealing at 250 °C. (**a**) 0 min, (**b**) 1 min, (**c**) 3 min and (**d**) 5 min.

Since the crystallization of the Se layer has been observed at 250 °C, it is worthwhile to look more closely at the time–dependent phase behavior of the Se layer at this temperature. Isothermal annealing time was varied from 0 to 5 min and the results of SEM and XRD analysis were compared in Fig. 3 wherein the intensities of XRD peaks were normalized by that of the Mo (110) peak at 2θ~40.5°. At 250 °C only part of the Se layer is crystallized, mainly around the surface. However, around 1 min of annealing at 250 °C seems likely to be sufficient to complete the crystallization of an entire 5 μm-thick Se layer. XRD results show that the intensity of the Se reflection peak is also maximized at this step, while that of the In peak remains unchanged. After 3 min at 250 °C, the top part of the crystalline Se layer begins to melt and yield an amorphous Se layer with a flat surface. After 5 min, the entire Se layer converts to a liquid phase and solidifies into amorphous layer after quenching, which is also evidenced by the total absence of Se reflection peaks in the XRD pattern.

It is also interesting to consider the behavior of the In XRD reflection peak. As displayed in Fig. 3, this peak remains unchanged until after 1 min of annealing at 250 °C, disappears after 3 min and then is recovered in the 5 min sample. It has been confirmed that this observed behavior of In is not an analytical artefact, as the results were repeatable. The equilibrium melting point of In and Se is around 156 °C and 221 °C, respectively. However, it is believed that the entirety of the In and some of the Se, is melted after just 3 min of annealing at 250 °C due to the rapid heating rate of 4 °C/s. During quenching after 3 min of isothermal annealing, liquid In is still covered by a crystalline Se layer and does not re-crystallize. In the case of Fig. 3(d) on the other hand, liquid In is mixed with liquid Se after 5 min at 250 °C; resulting in mostly amorphous Se forming during quenching and re-crystallization of the In, as evidenced by the recovery of the In XRD reflection peak. Therefore, it can be deduced that liquid or amorphous Se acts as a catalyst for the crystallization of In.

The results of isothermal experiments at 250 °C are particularly important to the design of low−temperature pre–annealing stages during the RTP of CuGaIn/Se type precursors. Song *et al*.^16^ used the 2-step selenization of a sputtered CuGaIn precursor, consisting of 10 min at 350 °C (1^st^ step) and 1–3 h at 550–650 °C (2^nd^ step) under Se vapor. They reported that this 2-step selenization resulted in the formation of liquid In-Se compounds and thus yielded a smoother surface morphology and a single phase of chalcopyrite. Similarly, Gremenok *et al*.^17^ also employed a 2-step selenization process, where the first step involved heating at 250–270 °C for 10–30 min and the second step involved heating at 460–540 °C for 10–50 min and subsequently obtained a chalcopyrite compound with a single phase and an improved morphology.

### 300 °C

**Figure 4:**
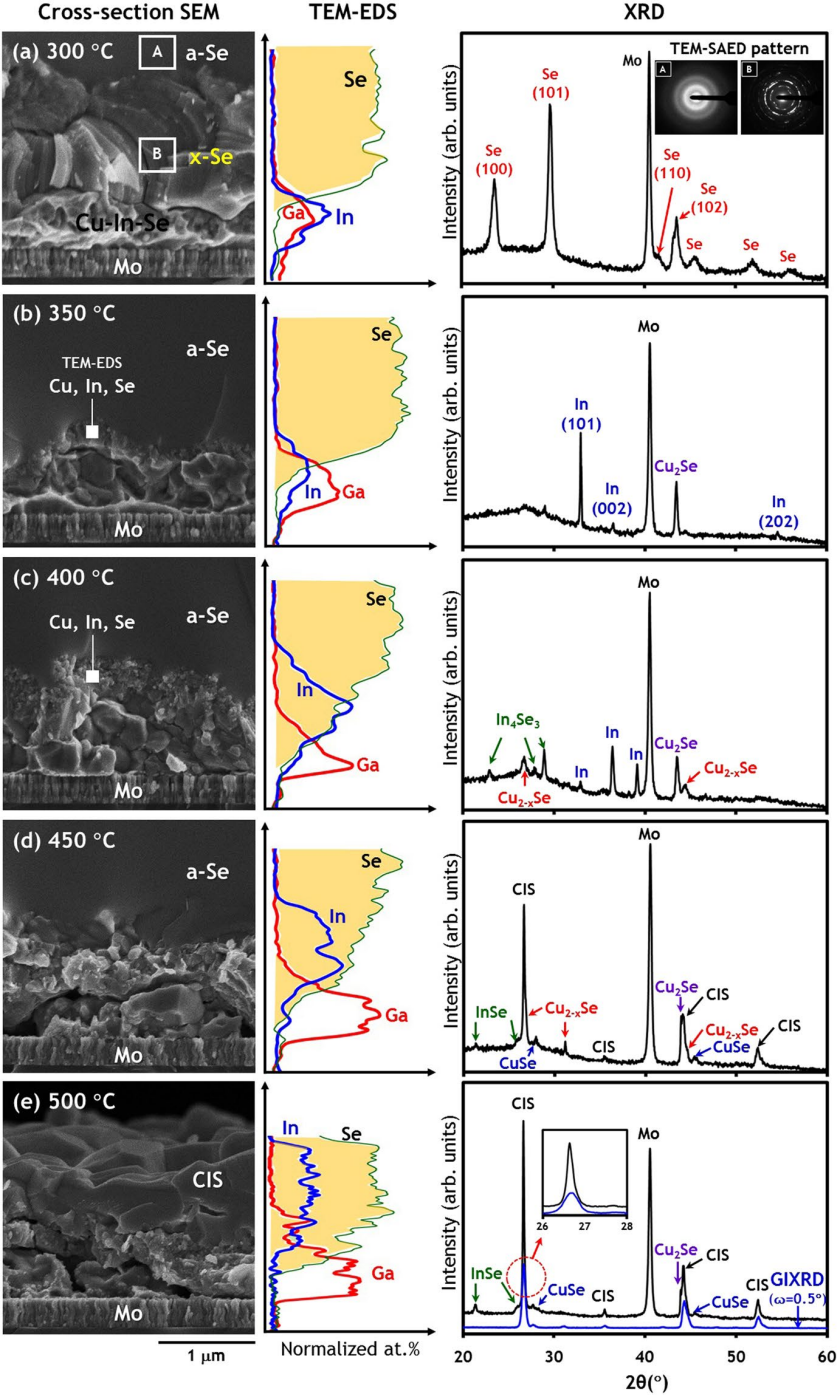
SEM images, TEM-EDS line scan and XRD scan results of samples quenched from 300–500 °C. (**a**) 300 °C, (**b**) 350 °C, (**c**) 400 °C, (**d**) 450 °C and (**e**) 500 °C. Inset SAED patterns in XRD chart of sample (**a**) were obtained from region [A] and [B] of cross-sectional SEM image. (**e**) GIXRD scan result with an incident angle ω = 0.5° was added to XRD chart for comparison.

The results of characterization for samples quenched from target temperatures of 300, 350, 400, 450 and 500 °C are summarized in Fig. 4(a–e), respectively (See also Supplementary Figure S3 for a wide range of SEM images). Figure 4(a) shows that the sample quenched from 300 °C has a similar structure to that obtained with the 250 °C, 3 min sample seen in Fig. 3(c), which is characterized by partial melting after complete crystallization of Se. The transmission electron microscopy (TEM)– selected-area electron diffraction (SAED) pattern confirmed the existence of an amorphous Se top layer and a polycrystalline Se bottom layer (see inset of Fig. 4(a) and Supplementary Figure S4). In reflection peaks are not detected, which is consistent with Fig. 3(c). The results of a TEM–energy dispersive X-ray spectroscopy (EDS) line scan demonstrate the separation of Se and metal layers with negligible interdiffusion. It should be noted that a Cu compositional profile is not reported here, since it is perturbed by the Cu TEM grid used in this analysis.

### 350 °C

As shown in Fig. 4(b), the crystalline Se layer is completely melted upon reaching a temperature of 350 °C and is subsequently solidified into an amorphous phase during quenching. The re-crystallization of the In phase is also confirmed by the XRD results, which are identical to the observed results for the 250 °C, 5 min sample shown in Fig. 3(d). Additionally, the XRD results suggest the formation of a Cu–rich Cu_2_Se phase. A 200~300 nm–thick layer is produced at the interface of intermetallic Cu–Ga–In and Se layers, as shown in the SEM image; and has been confirmed to consist of Cu, In and Se, as characterized by TEM–EDS spot analysis. The TEM–EDS line scan also suggests a slight interdiffusion of In and Se.

### 400 °C

In this stage, the metallic Cu, In and Se elements have actively diffused into each other, whilst Ga remains at the bottom owing to its poor mobility and relatively lower reactivity with Se compared to In. The formation of Se–poor binary phases such as In_4_Se_3_, Cu_2-x_Se was also identified. It is interesting to note that unreacted liquid In crystallizes predominantly in the (002) orientation, which differs from those samples quenched from temperatures of 350 °C or less where the In (101) peak was dominant.

### 450 °C

At this temperature CIS is finally formed, with more Se incorporated into Se-poor binaries to form CuSe and InSe phases. The separation of In and Ga becomes more pronounced, resulting in In–containing compounds (e.g., InSe and CIS) in the upper layer and a Ga–containing amorphous intermetallic at the bottom.

### 500 °C

At this stage CIS crystal grains continue to grow, as evidenced by the sharp and strong CIS reflection peaks in Fig. 4(e). However, several binary selenides are still detected, which means the formation of the chalcopyrite structure is not yet complete. Large grains in the upper region of the cross–sectional SEM image are believed to be mainly chalcopyrite CIS crystals, as confirmed by grazing incidence XRD (GIXRD) measured with a shallow incident angle of ω = 0.5°. Based on the TEM–EDS depth profile, there appears to be a bottom layer around 250 nm in thickness that is likely to consist of a Cu–Ga intermetallic without any In or Se. This is a result of the fact that In diffuses towards the surface and the rate of Se diffusion is not sufficient to reach the bottom layer. Additionally, it should be noted that the Se layer on the surface of the sample is completely depleted at 500 °C, primarily due to vaporization of Se caused by its high vapor pressure and reaction with Cu–In–(Ga) precursors. Loss of the 5 μm–thick Se layer is initiated at around 350 °C and completed by 500 °C (see Supplementary Figure S5). At temperatures in excess of 500 °C, further selenization and crystallization will proceed by virtue of Se vapor still trapped within the covered sample tray.

### 550 °C (Isothermal annealing)

**Figure 5:**
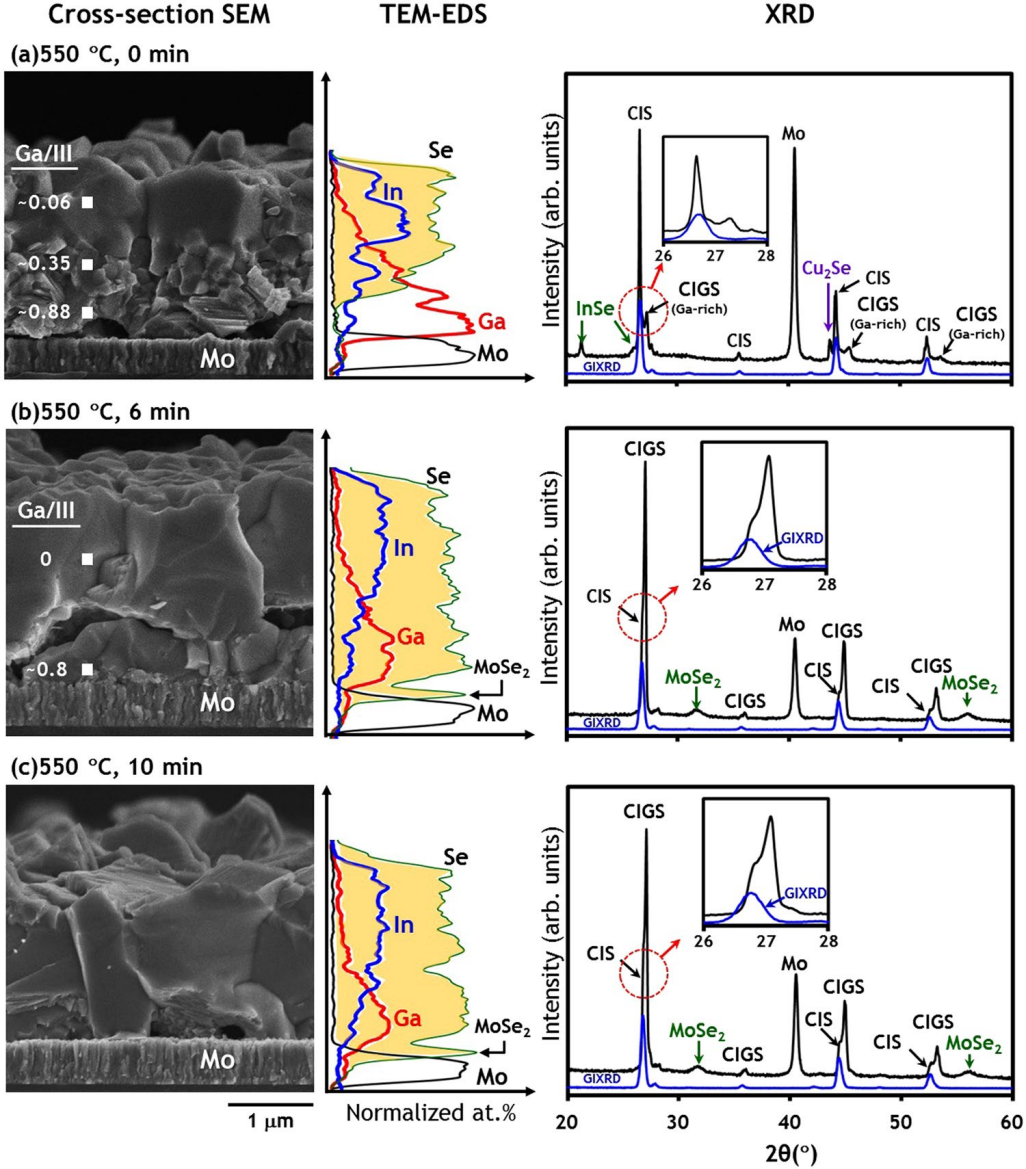
SEM images, TEM-EDS line scan, XRD (with GIXRD) scan results of samples quenched from isothermal annealing at 550 °C. (**a**) 0 min, (**b**) 6 min and (**c**) 10 min. Spot TEM-EDS composition results were added to SEM images. GIXRD scan results were obtained with an incident angle ω = 0.5°.

As shown in Fig. 5, a temperature of 550 °C is enough for Ga to have sufficient mobility to allow it to be incorporated into a chalcopyrite structure and thus CIGS reflection peak begins to grow whilst binary InSe and Cu_2_Se peaks remain detectable. GIXRD results show that CIS or In-rich CIGS is located in the upper layer, with Ga–rich CIGS at the bottom, which is also evidenced by TEM–EDS line scan and spot analysis results. A close look at the TEM–EDS line scan results reveals that Se does not yet reach the Mo surface and unreacted Cu–Ga intermetallic phases still exist at the bottom, suggesting that selenization of the metallic precursor is still not complete.

**Figure 6:**
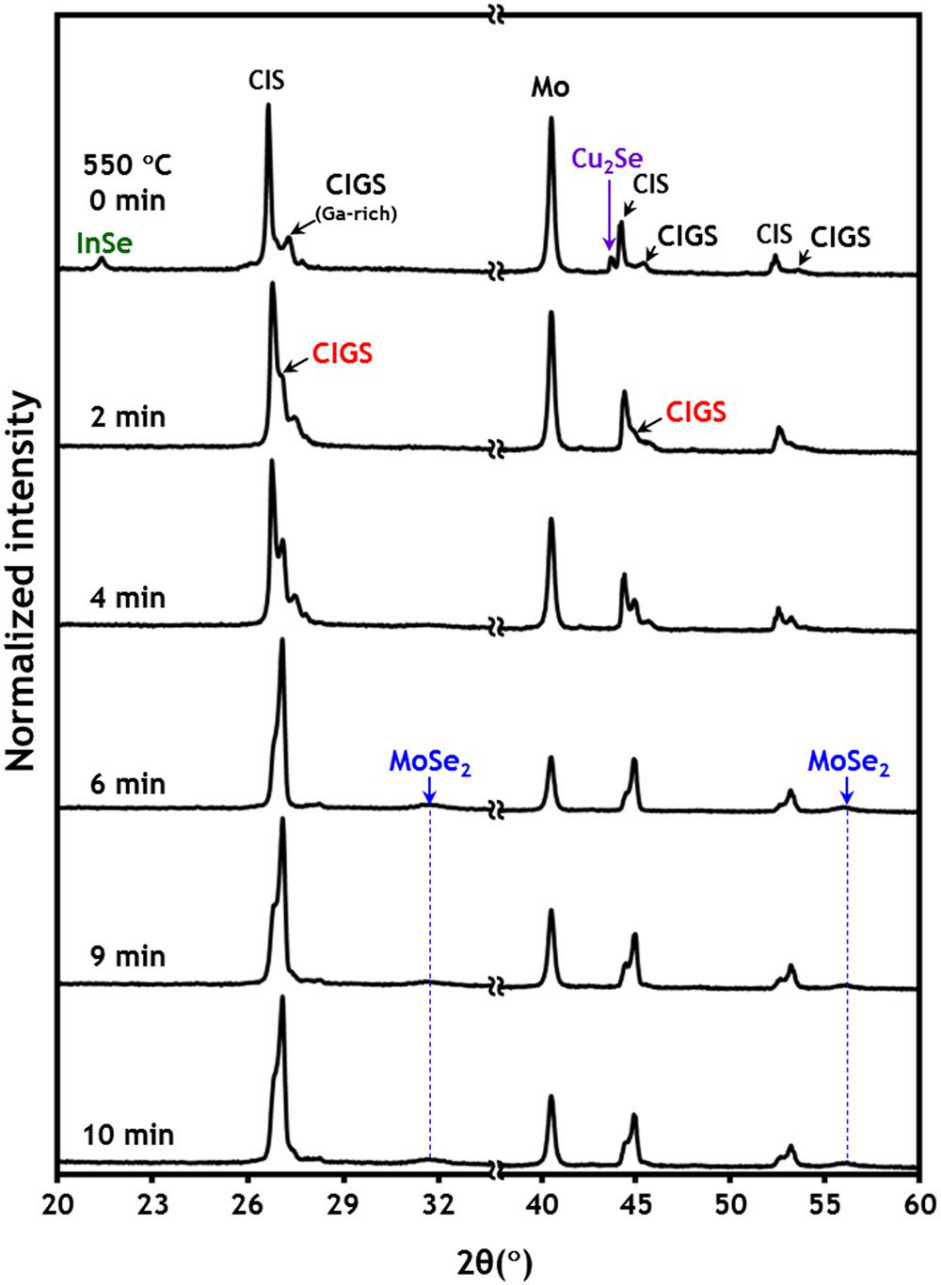
XRD scan results of samples quenched after isothermal annealing at 550 °C. Isothermal time is 0, 2, 4, 6, 9 and 10 min.

Consequently, a series of isothermal annealings from 550 °C was performed for 0, 2, 4, 6, 9 and 10 min. The incorporation of Ga into the CIGS phase was enhanced with annealing time at a fixed temperature of 550 °C, as evidenced by the XRD patterns (see Fig. 6). After only 2 min of isothermal annealing, all binary intermediate phases had disappeared. The formation of MoSe_2_ at 6 min indicated that the reaction had proceeded to completion^15,18^. The formation of MoSe_2_ was confirmed by a spike of Se on the surface of Mo in a TEM–EDS line scan, as well as in the XRD pattern of Fig. 5(b). The sample obtained after 10 min of isothermal annealing, in Fig. 5(c), was not significantly different from that after 6 min and most notably did not show any further improvement in the uniformity of Ga with depth. It is quite promising to discover that CuGa/In/Se precursors can be completely converted to CIGS within 6 min at 550 °C and with a ramp rate of 4 °C/s, as time is one of the critical issues in the scaling–up of 2–step precursor–selenization methods.

## Conclusions

**Figure 7:**
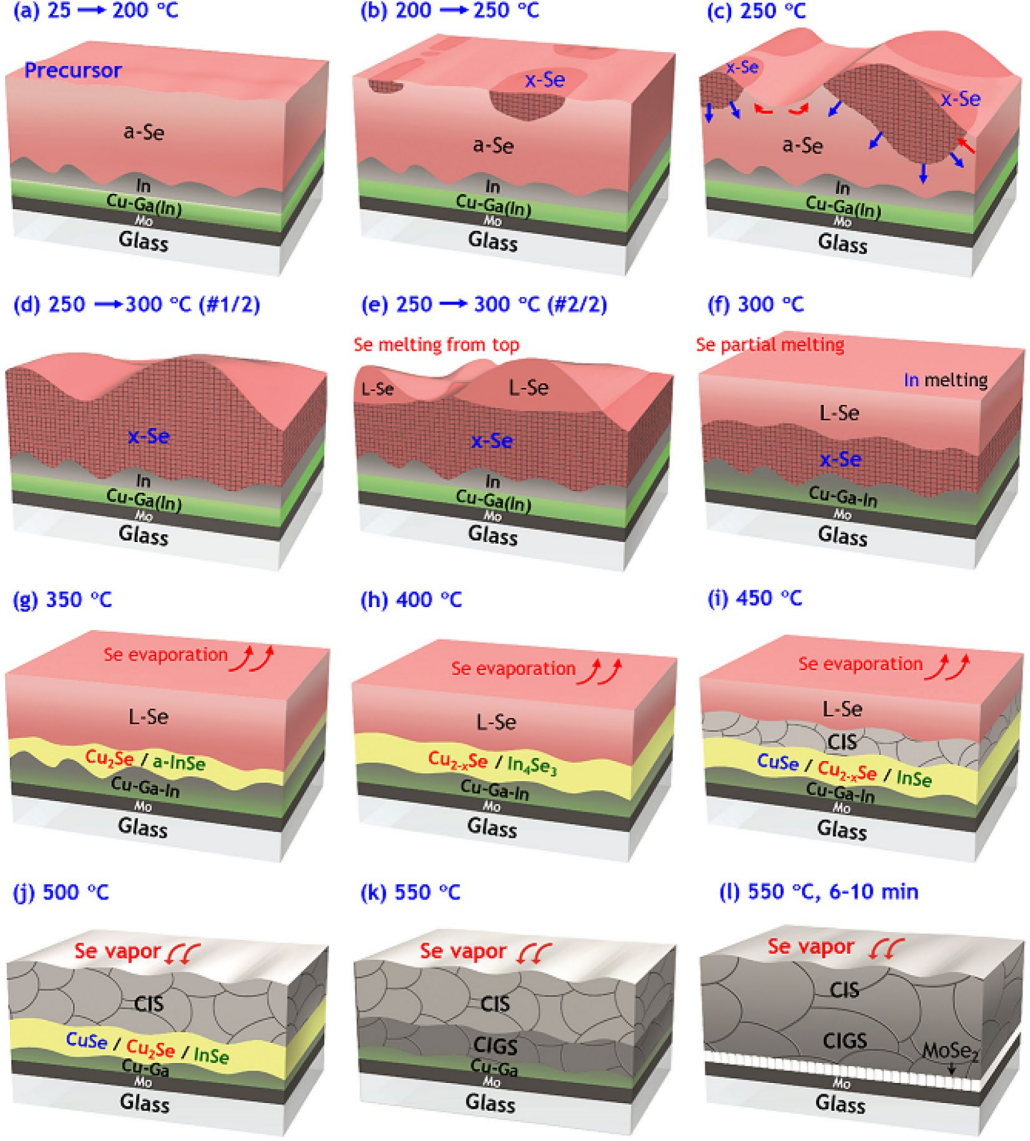
Visualized cartoon for detailed phase evolution during RTP of glass/Mo/CuGa/In/Se precursor with a ramp rate of 4 °C/s.

A detailed look at the thermal behaviour of a Se layer and CuGaIn intermetallic phases was achieved through systematic investigation by a diverse range of characterization techniques. On the basis of experimental results and thermodynamic equilibrium information in the literature, we propose an overall mechanism for the phase evolution of a CuGa/In/Se precursor, as illustrated in Fig. 7. This visualization of the selenization pathways can be effectively utilized by both researchers and engineers to understand what actually occurs during the RTP of Se-coated bilayer CuGa/In and similar precursors, in order to better optimize the process and precursor structures. In particular, the information regarding the crystallization and melting of Se from isothermal runs at 250 °C is directly relevant to the design of the pre-annealing stage (e.g., annealing temperature, ramp rate and time). This is critical for obtaining high-quality chalcopyrite crystals and reliable adhesion of CIGS to Mo, while also preventing damage to the glass substrate.

## Methods

### Precursor preparation

Precursors were prepared from 1.8 mm thick low-alkali, high-strain-point glass (PV200, Asahi glass) with a 300 nm Mo coating. These were subsequently coated using DC-magnetron sputtering of CuGa (24 Ga wt.%) alloy at a power of 5 W/cm^2^ and pure In targets at 2.5 W/cm^2^, yielding an overall dynamic deposition rate of approximately 100 nm/min. A Se layer with a thicknesses of c.a. 5 μm was then deposited at a rate of 1–1.2 nm/sec onto the sputtered CuGa/In bilayer by evaporating a Se source using an effusion cell with a pyrolytic boron nitride crucible in a high-vacuum (~10^−6^ Torr) evaporator. The thickness of the Se layer was confirmed in cleaved samples by using FE-SEM(Hitachi S-4800). The precursor was intended to have a total thickness of 600 nm and a preferred atomic composition of Cu/III = 0.90~0.91 and Ga/III = 0.30~0.31.

### Quenching experiments

The glass/Mo/CuGa/In/Se precursors were thermally treated under an ambient Ar atmosphere based on the pre-set time-temperature profile (Supplementary Figure S3) of a RTP system. The equipment used consisted of a quartz tube reactor 62 mm in diameter, a quartz sample tray, an infrared heater surrounding the tube and a cold zone wrapped by a Cu cooling coil. To minimize unnecessary Se loss, precursors were enclosed in the quartz tray by a transparent quartz cover. Further details of the RTP system and operation protocol can be found in our previous paper^12^. In a series of experiments, as shown in the time-temperature profile (Supplementary Figure S6), samples were heated at a rate of 4 °C/s and then quenched by the following cooling procedure: Samples are heated until the temperature reaches the target value, then the lamp heaters are immediately turned off and the samples are transferred to a cooling zone wrapped by a Cu cooling coil. At the same time, the flow rate of the Ar purge gas is dramatically increased from 100 to 2,000 sccm.

### Sample characterization

Samples obtained during rapid heating to 550 °C and isothermal annealing at 250 and 550 °C were systematically analysed by several characterisation techniques: XRD (PANalytical X’pert Pro MPD) and GIXRD with CuK_α1_ incident radiation, FE-SEM with an acceleration voltage of 5 kV, high-resolution TEM (Hitachi HF-3300 operated at 300 kV) and SAED patterns. The thin samples for TEM analysis were fabricated by a focused ion beam (Hitachi NB 5000). The compositional depth profile and atomic composition at specific positions within each cleaved sample were estimated using TEM-EDS.

## Electronic supplementary material

Supplementary figures

## References

[1] Kamada, R. *et al*. New world record Cu(In, Ga)(Se, S)_2_ thin film solar cell efficiency beyond 22%, *IEEE Xplore Digital Library*. 10.1109/PVSC.2016.7749822 (2016).

[2] Kushiya K (2008). Interface control to enhance the fill factor over 0.70 in a large-area CIS-based thin-film PV technology. Thin Solid Films.

[3] Kushiya K (2009). Key near-term R&D issues for continuous improvement in CIS-based thin-film PV modules. Sol. Energy Mater. Sol. Cells.

[4] Avancis produces champion CIGS module with 17.9% conversion efficiency. *Mark Osborne*. https://www.pv-tech.org/news/avancis-produces-champion-cigs-module-with-17.9-conversion-efficiency (2016).

[5] Karg F (2012). High efficiency CIGS solar modules. Energy Procedia.

[6] Caballero R, Guillén C (2005). CuInSe_2_ formation by selenization of sequentially evaporated metallic layers. Sol. Energy Mater. Sol. Cells.

[7] Abou-Ras D (2005). Formation and characterization of MoSe_2_ for Cu(In,Ga)Se_2_ based solar cells. Thin Solid Films.

[8] Wurz R (2003). Formation of an interfacial MoSe layer in CVD grown CuGaSe_2_ based thin film solar cells. Thin Solid Films.

[9] Nishiwaki S (1998). MoSe_2_ layer formation at Cu(In,Ga)Se_2_/Mo Interfaces in High Efficiency Cu(In_1- x_Ga_x_)Se_2_ Solar Cells. Jpn. J. Appl. Phys..

[10] Zhang, Z., Kobayashi, M. & Yamada, A. Comparison of Ag(In,Ga)Se_2_/Mo and Cu(In,Ga)Se_2_/Mo Interfaces in Solar Cells. *ACS Appl. Mater. Interfaces* **9**, 16215–16220 (2017). 10.1021/acsami.7b02548 28448114

[11] Mueller BJ (2016). Influence of Mo–N as diffusion barrier in Mo back contacts for Cu(In,Ga)Se_2_ solar cells. Thin Solid Films.

[12] Lima JC, Grandi TA, Biasi RS (2001). Influence of aging on the thermal and structural properties of amorphous selenium prepared by ball milling. Journal of Non-Crystalline Solids.

[13] Zhang W, Ma F, Zhang T, Xu K (2011). Stress and microstructure evolution in Al-induced crystallization of amorphous Ge thin films. Thin Solid Films.

[14] Leervad Pedersen TP (2001). Mechanical stresses upon crystallization in phase change materials. Appl. Phys. Lett..

[15] Koo J (2013). Optimization of Se layer thickness in Mo/CuGa/In/Se precursor for the formation of Cu(InGa)Se_2_ by rapid thermal annealing. Thin Solid Films.

[16] Song HK (2003). Fabrication of CuIn_1−x_Ga_x_Se_2_ thin film solar cells by sputtering and selenization process. Thin Solid Films.

[17] Gremenok VF (2005). Preparation of Cu(In,Ga)Se_2_ thin film solar cells by two-stage selenization processes using N_2_ gas. Sol. Energy Mater. Sol. Cells.

[18] Kim WK, Payzant EA, Yoon S, Anderson TJ (2006). *In situ* investigation on selenization kinetics of Cu–In precursor using time-resolved, high temperature X-ray diffraction. J. Cryst. Growth..

