# Supplementary material for: Detailed Visualization of Phase Evolution during Rapid Formation of Cu(InGa)Se2 Photovoltaic Absorber from Mo/CuGa/In/Se Precursors
Source: Sci Rep. 2018 Mar 2;8:3905. doi: 10.1038/s41598-018-22214-y (PMC5834449; doi:10.1038/s41598-018-22214-y)
Supplement: Supplementary file 1 — Supplementary figures [file 41598_2018_22214_MOESM1_ESM.pdf]

Supplementary information

# Detailed Visualization of Phase Evolution during Rapid Formation of $Cu(InGa)Se_2$ Photovoltaic Absorber from Mo/CuGa/In/Se Precursors

Jaseok Koo[1], Sammi Kim[1], Taewoon Cheon[2], Soo-Hyun Kim[3] & Woo Kyoung Kim[1,*]

[1] School of Chemical Engineering, Yeungnam University, Gyeongsan, Gyeongbuk, 38541, Republic of Korea
[2] Daegu Gyeongbuk Institute of Science & Technology, Dalseong, Daegu, 42988, Republic of Korea
[3] Department of Material Science and Engineering, Yeungnam University, Gyeongsan, Gyeongbuk, 38541, Republic of Korea

[*] Corresponding author: Prof. Woo Kyoung Kim, address: School of Chemical Engineering, Yeungnam University, Gyeongsan, Gyeongbuk 38541, Republic of Korea, tel.: 82-53-810-2514, fax: 82-53-810-4631, e-mail: wkim@ynu.ac.kr

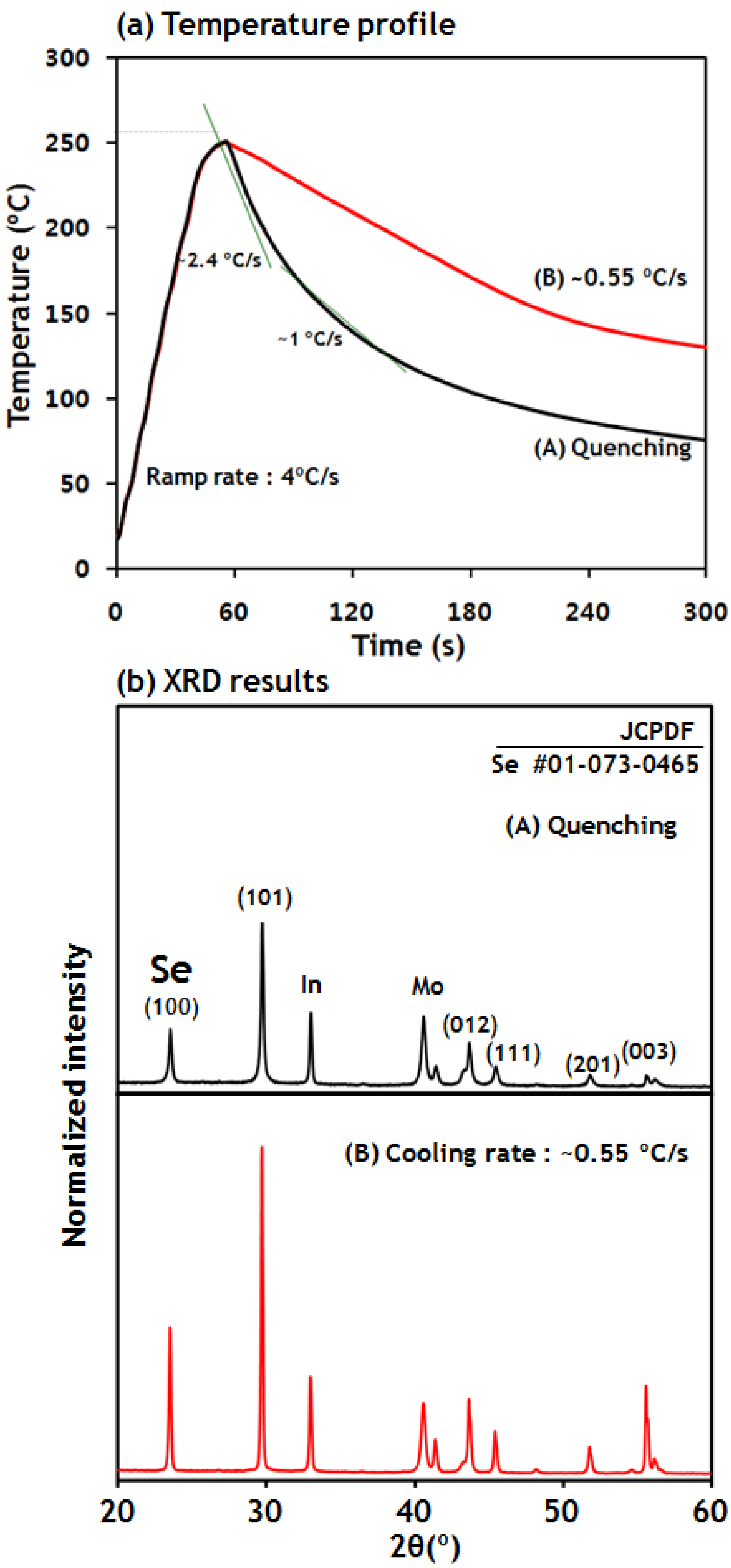


Figure S1. (a) Tempeature profile with different cooling rates and (b) XRD scan results of samples quenched from 250 °C with different cooling rates. (A) quenching and (B) -0.55 ℃/s. XRD peak intensities were normalized by that of the Mo (110) peak at 2θ~40.5°

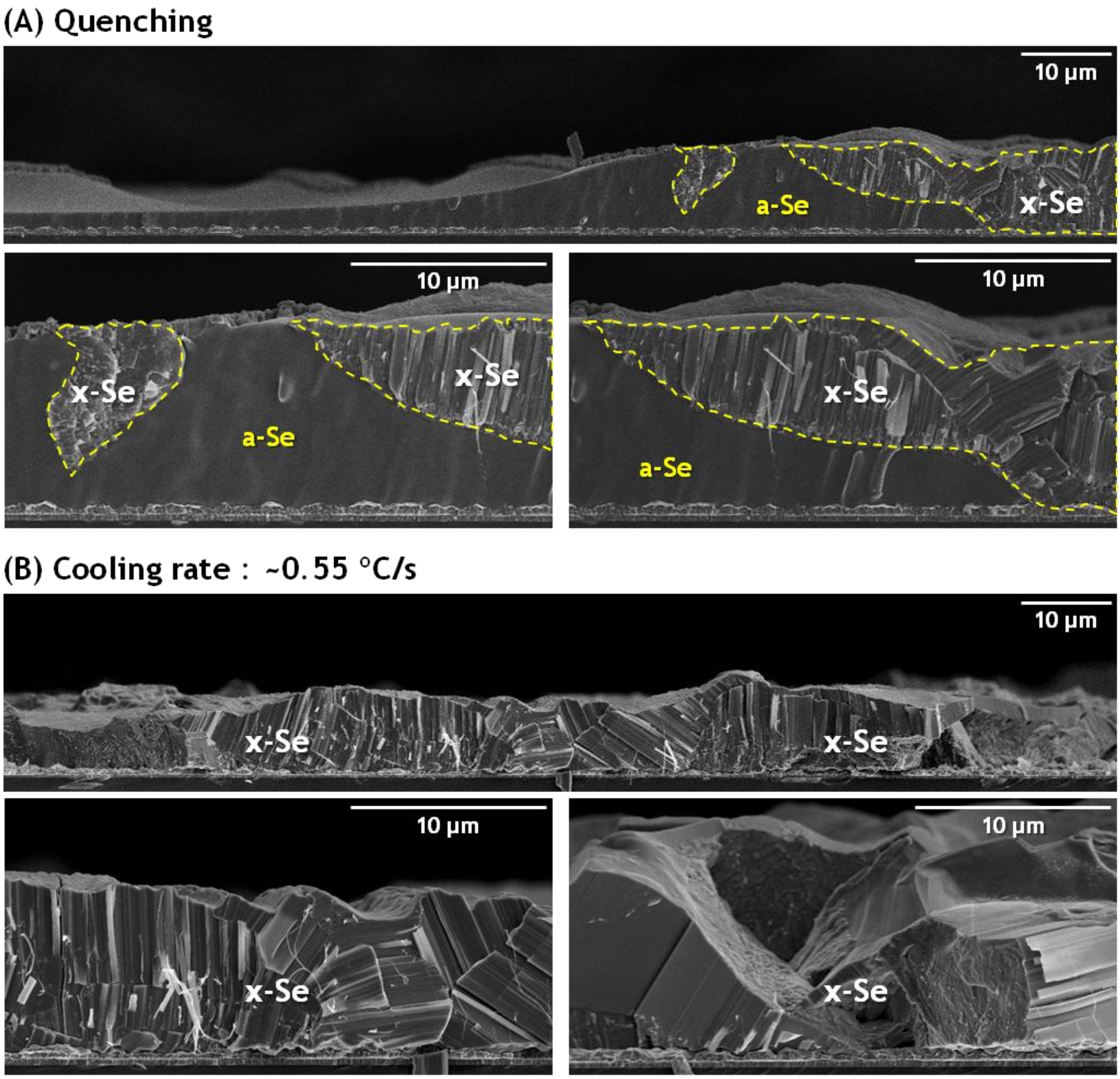


Figure S2. Cross-sectional SEM images of samples quenched from 250 °C with different cooling rates. (A) quenching and (B) - 0.55℃/s..

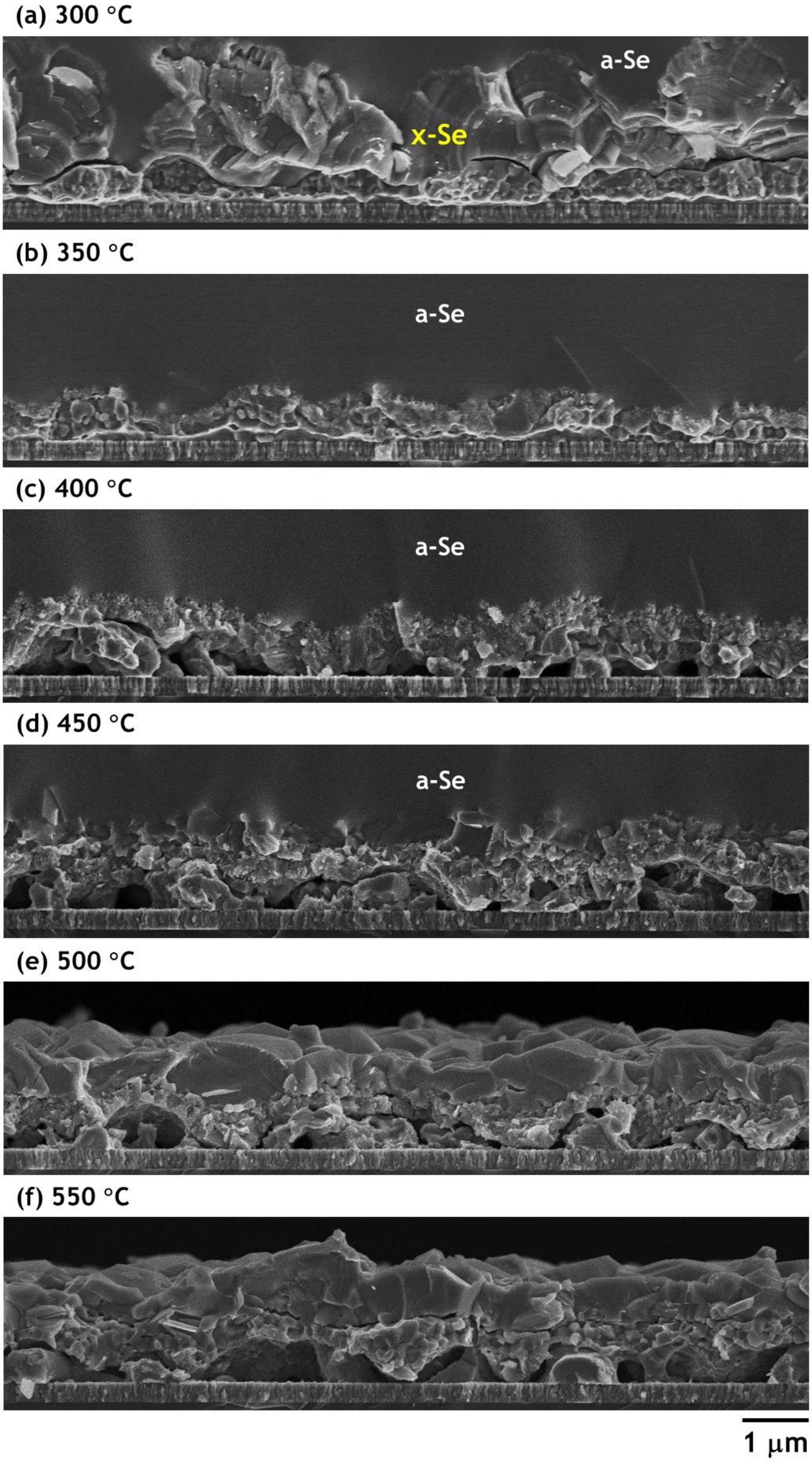


Figure S3. SEM cross-sectional images of samples quenched from 300-550 °C. (a) 300 °C, (b) 350 °C, (c) 400 °C, (d) 450 °C, (e) 500 °C and (f) 550 °C

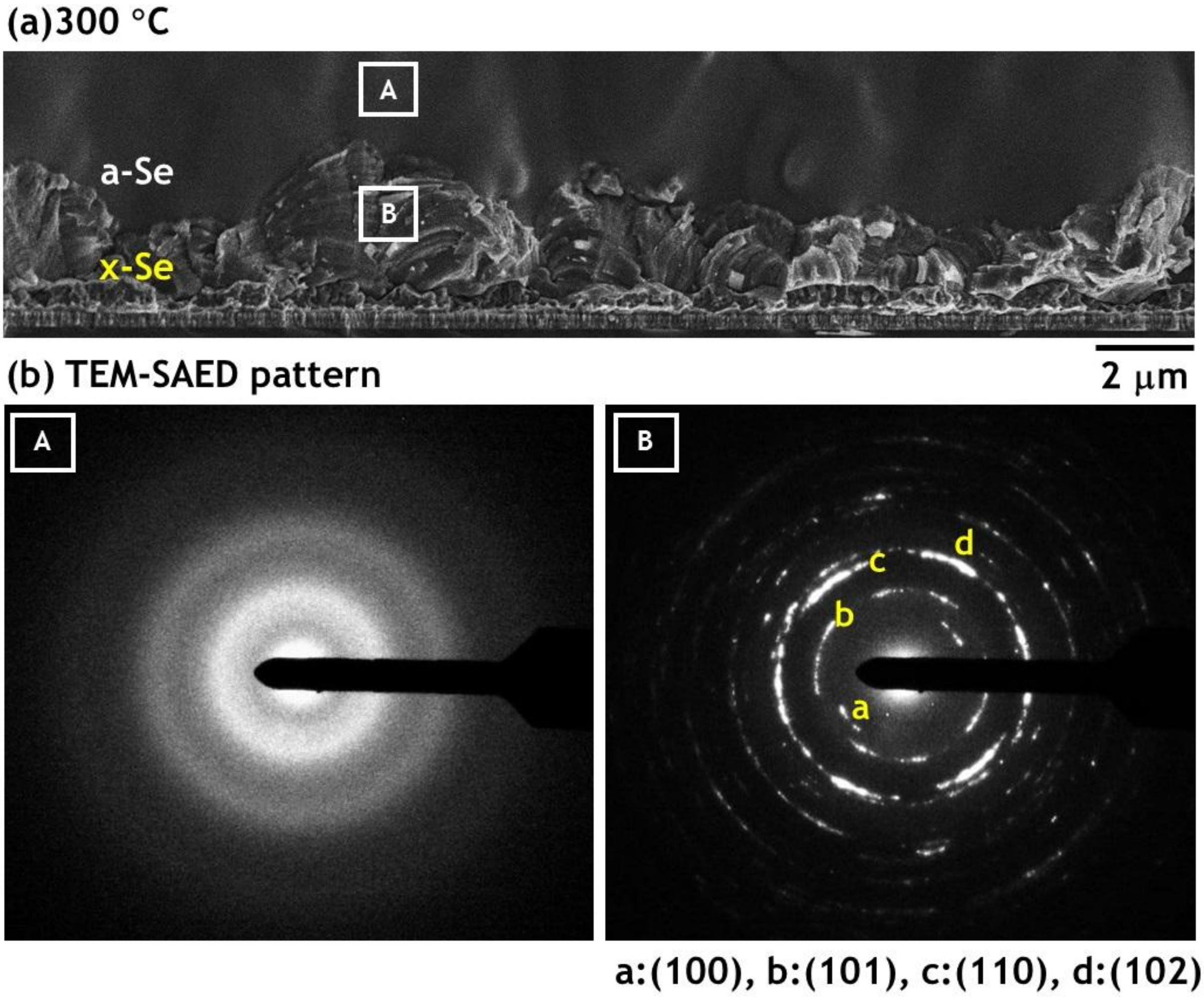


Figure S4. (a) SEM cross-sectional image and (b) TEM-SAED patterns of sample quenched from 300 °C

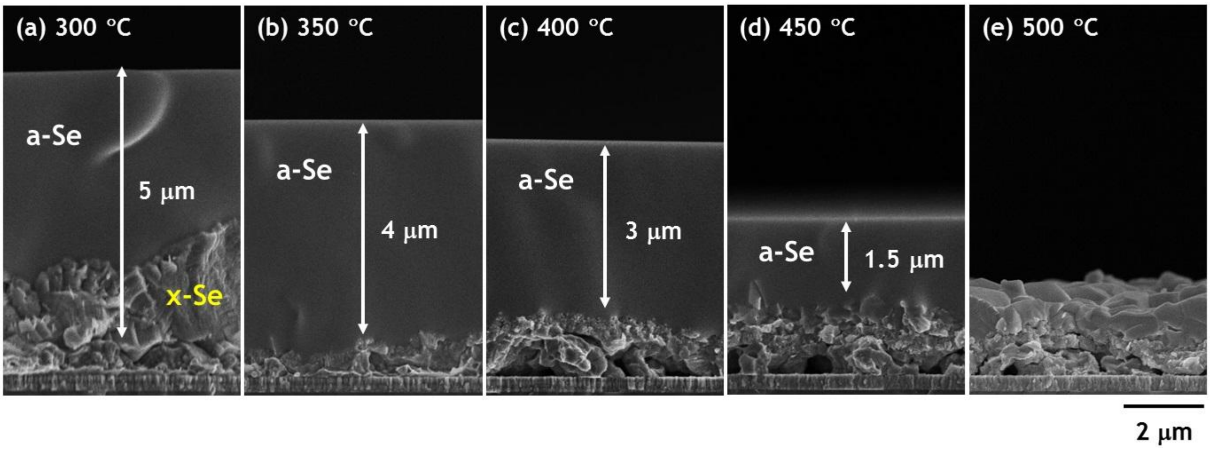


Figure S5. Variation of Se layer thickness with temperature : (a) 300 °C, (b) 350 °C, (c) 400 °C, (d) 450 °C and (e) 500 °C.

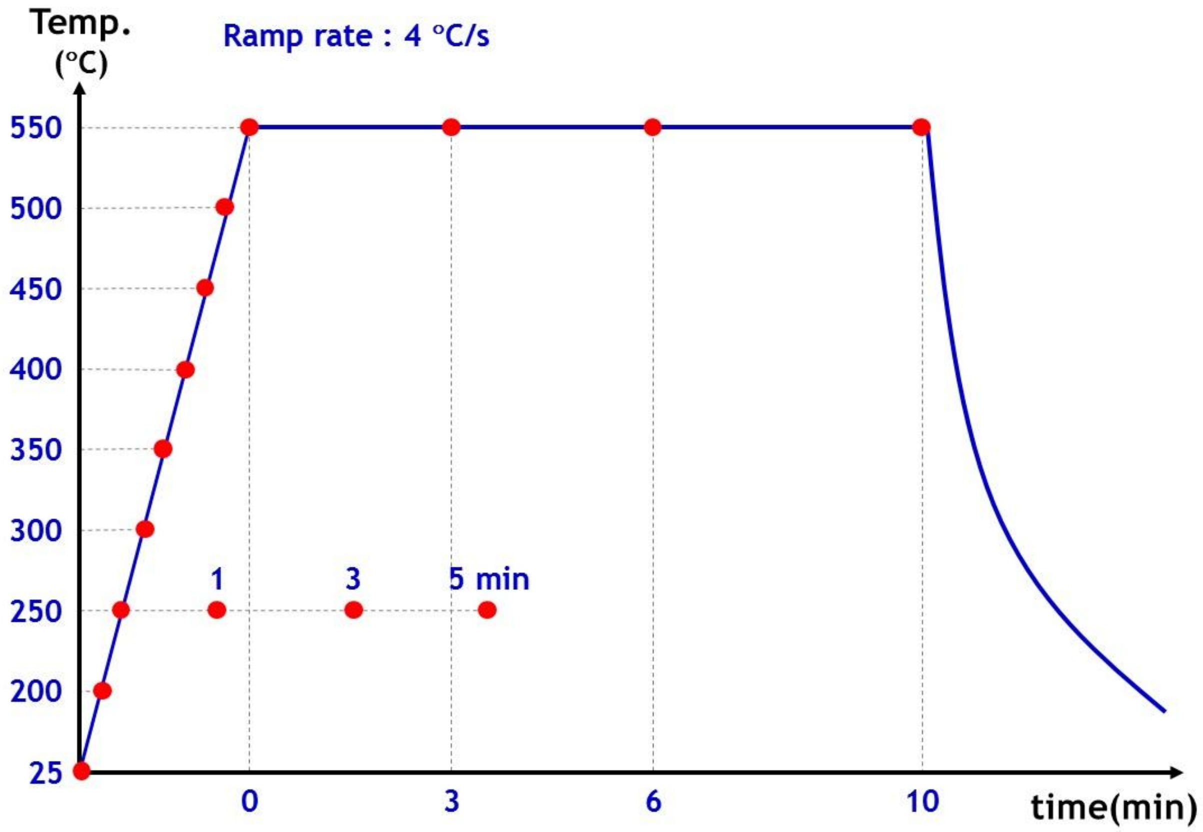


Figure S6. Time-temperature profile used for sampling in the quenching experiments.
